# Effects of Sport-Based Interventions on Children’s Executive Function: A Systematic Review and Meta-Analysis

**DOI:** 10.3390/brainsci11060755

**Published:** 2021-06-07

**Authors:** Falonn Contreras-Osorio, Christian Campos-Jara, Cristian Martínez-Salazar, Luis Chirosa-Ríos, Darío Martínez-García

**Affiliations:** 1 Department Physical Education and Sports, Faculty of Sport Sciences, University of Granada, 18011 Granada, Spain; falonn.contreras@unab.cl (F.C.-O.); lchirosa@ugr.es (L.C.-R.); 2 Faculty of Rehabilitation Sciences, Universidad Andres Bello, Santiago de Chile 7591538, Chile; 3 Departamento de Educación Física, Deportes y Recreación, Pedagogía en Educación Física, Facultad de Educación y Ciencias Sociales y Humanidades, Universidad de La Frontera, Temuco 4780000, Chile; cristian.martinez.s@ufrontera.cl; 4 Faculty of Rehabilitation Sciences, Universidad Andres Bello, Concepción 4030000, Chile; damaga1991@gmail.com

**Keywords:** executive function, inhibitory control, working memory, cognitive flexibility, sport, children

## Abstract

One of the most studied aspects of children’s cognitive development is that of the development of the executive function, and research has shown that physical activity has been demonstrated as a key factor in its enhancement. This meta-analysis aims to assess the impact of specific sports interventions on the executive function of children and teenagers. A systematic review was carried out on 1 November 2020 to search for published scientific evidence that analysed different sports programs that possibly affected executive function in students. Longitudinal studies, which assessed the effects of sports interventions on subjects between 6 and 18 years old, were identified through a systematic search of the four principal electronic databases: Web of Science, PubMed, Scopus, and EBSCO. A total of eight studies, with 424 subjects overall, met the inclusion criteria and were classified based on one or more of the following categories: working memory, inhibitory control, and cognitive flexibility. The random-effects model for meta-analyses was performed with RevMan version 5.3 to facilitate the analysis of the studies. Large effect sizes were found in all categories: working memory (ES −1.25; 95% CI −1.70; −0.79; *p* < 0.0001); inhibitory control (ES −1.30; 95% CI −1.98; −0.63; *p* < 0.00001); and cognitive flexibility (ES −1.52; 95% CI −2.20; −0.83; *p* < 0.00001). Our analysis concluded that healthy children and teenagers should be encouraged to practice sports in order to improve their executive function at every stage of their development.

## 1. Introduction

The executive function is a set of mental processes that enables the regulation of thoughts and actions during goal-directed behaviour [1]. It is also responsible for monitoring and controlling the mechanisms that mediate the use of information [2]. There is a consensus for the classification of executive function into three main capacities: inhibition, working memory, and cognitive flexibility [3,4]. Inhibition relates to attention and behaviour and prevents our acting under the control of irrelevant environmental stimuli. This allows humans to resist distractions from the environment or memory [1,5]. Working memory makes it possible to hold information in order to perform cognitive operations, relating information, and integrating it into an appropriate response [6]. There are two types of working memory depending on their nature: verbal working memory and non-verbal (visual–spatial) working memory [7]. Cognitive flexibility is the ability to adjust our behaviour appropriately and efficiently according to changes in the environment [8]. In this way, cognitive flexibility interacts with the other components of executive function to respond to the new demands of the proposed task [6,8].

The development of the executive function during childhood and adolescence has been one of the most studied aspects of cognitive growth. It enhances the ability to respond to environmental demands, influencing physical health, school success, cognitive, social, and psychological development [6]. The development of this ability in children and adolescents is associated with physical activity and sport [9,10,11,12,13,14,15,16,17]. This issue has been studied based upon athletic performance, focusing on the identification of variables that improve success at the competitive level [16,18,19,20,21,22,23]. Although sports and physical activity in a school context, in particular, sports with changing situations that require anticipating and making decisions considering multiple variables dynamically and flexibly, has been hypothesised to be beneficial for children’s development, it has not been widely studied [24,25,26,27,28,29,30,31].

Sport is an activity involving physical movement, with defined goals, containing explicit formal rules and structured relationships between athletes [32]. There are multiple health benefits for children and adolescents, such as psychological, cognitive, social [14,18,33,34,35,36,37,38,39,40,41,42], and musculoskeletal development [43,44,45,46,47], reduced risk of suffering obesity or being overweight [48,49,50], and improved quality of life [40,51,52]. The impact of sport on brain function is significant as it is considered to promote social development and integration, contributing to well-being, even in adulthood [53,54].

The mechanisms linking exercise and cognitive function can be classified into two broad categories: physiological and learned [55]. Physiologically, aerobic exercise may enhance neuronal activity, monoaminergic transmission, neurotrophic signalling, and processes that mediate neuroplasticity [55,56,57,58]. Acute physical activity has been found to raise physiological arousal in children and adolescents, resulting in increased cerebral blood flow and neuronal connectivity. Cognitive efficiency is facilitated through higher attention allocation and improvements in information processing [55,56]. Intervention programs involving continuous aerobic physical activity over several weeks create a metabolic and structural brain environment that supports the development of cognitive functions and learning processes, such as synapses, neuroglia, myelination, vascularisation, and the expression of genes and growth factors related to brain plasticity, among others [55,57]. Brain changes resulting from aerobic exercise have been shown to benefit cognitive function in healthy children and adolescents with attention deficit hyperactivity disorder (ADHD) [59].

Learning-related mechanisms focus on the cognitive demand that exists during the learning of motor skills and the coordination of complex movements, enhancing decision-making processes, or flexible behaviour in response to demands [15,60,61]. A combination of both mechanisms during sport (with an aerobic training aspect) may impact the development of cognitive activities, having a more significant effect on executive functioning [55,62,63,64]. Additionally, it is important to include elements that enhance the emotional, social, and character development of children, with progressively more challenging activities that take advantage of their interests and motivation [64,65].

Understanding and interpreting the relationship between these variables has improved through the study and exploration of brain changes derived from sports practice [36]. Techniques such as functional magnetic resonance imaging and electroencephalography have been used [66,67,68]. Functional magnetic resonance imaging is a measurement of small metabolic changes in brain regions related to executive function during exercise or the performing of tasks [36,66]. Electroencephalography aims to determine the location of the electrical activity generated in the human brain by identifying the source of the signal to a specific set of neurons, which is possible by isolating interference [69]. These techniques provide the opportunity to identify differences in brain activity during certain mental tasks involving executive functions, such as creative thinking and decision-making in different situations [68]. These techniques are complementary to neuropsychological exploration through the use of direct (on the child or teenager) or indirect (mainly aimed at parents or teachers) tests, which vary in terms of test format (e.g., pen and paper, computer), administration time, type of administration (individual or group), reliability, and validity [6,70].

Previous systematic reviews and meta-analyses have analysed the effects of acute and chronic physical activity interventions on cognitive outcomes in children and adolescents, finding positive outcomes for various cognitive skills, such as processing speed, attention, and language, among others [18,71,72,73,74,75,76]. These studies, focused on non-regulated physical activity, show improvements to a small and moderate extent in terms of general executive function, or at least in one of its domains [9,10,11,12]. Whereas the previous literature suggests that higher levels of physical activity can positively affect executive function, not enough research has been conducted to differentiate between the respective impacts of physical activity, exercise, and sport [18]. Differences in the characteristics of these modalities might cause different changes in the development of executive function in children and teenagers [10,12].

Therefore, the present meta-analysis aims to: (I) collect as much scientific evidence as possible to conduct a qualitative analysis of the methodology of studies focusing on the influence of sports interventions on children’s executive function and (II) analyse the effect of sports programs on executive function, as a whole or in any of its three main dimensions, in healthy children and teenagers.

## 2. Materials and Methods

**Figure 1:**
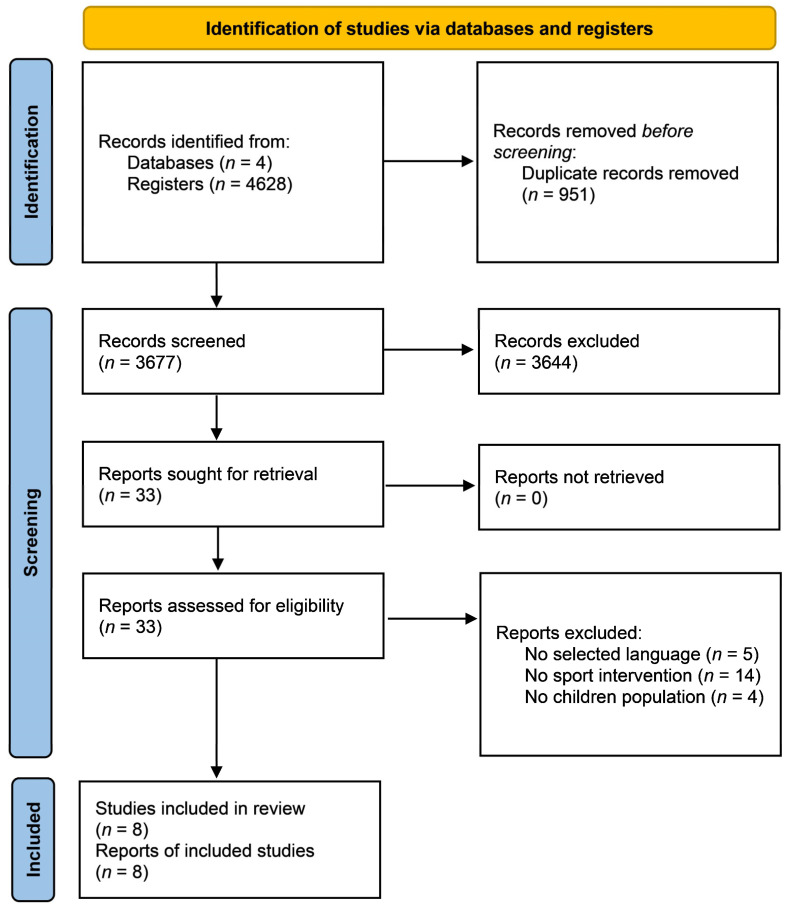
A flow diagram of the studies included in the meta-analysis.

A systematic review was carried out on 1 November 2020 to search the published scientific evidence in order to understand how different sports programs affected executive function in students. The reporting flow diagram of this systematic review was based on the preferred reporting items for systematic reviews and meta-analyses (PRISMA) guidelines [77] (Figure 1).

### 2.1. Search Strategy

Longitudinal design studies were identified by searching the four principal electronic databases: Web of Science, SCOPUS, EBSCO, and PubMed. The bibliographic search was carried out by combining the different medical subject heading (MeSH) terms with the following keywords: “Executive Functions”, “Cognitive Functions”, Cognition, “Executive Control”, “Cognitive Functioning”, “Cognitive Control”, “Sports”, “Athletics”, “Sport Practice”, “Sport Performance”, and “Physical Activity”. These search terms were combined with two Boolean operators: AND OR. Additionally, the bibliographies of other previous related reviews and the studies ultimately selected were examined to search for new studies. Other possible scientific evidence related to the subject was identified by contacting the authors of the published articles via email (Table S1).

Two independent reviewers (FCO and CCJ) examined the title and summary of the articles found in the databases. After the initial selection, they analysed each study with the inclusion criteria. Each criterion was evaluated as yes or no. If discrepancies existed between the authors, the ratings of the articles were shared and discussed until a consensus was reached with a third reviewer (DMG). The authors were familiar with the existing literature and were not biased toward any of the studies selected for inclusion in the review.

### 2.2. Inclusion Criteria

The eligibility of each investigation was measured according to the following inclusion criteria: (a) longitudinal studies with, at least, an experimental group (pre- and post-intervention design); (b) the subjects of the study had to be healthy children (age 6–12 years) and teenagers (age 13–17 years); (c) chronic sport-based interventions (an intervention program that involves sports activities over several days) supervised by an expert; (d) executive function or one of its main categories had to appear in the study’s reported data; (e) the study had to have sufficient data to calculate effect sizes (ES). The articles that met the inclusion criteria were identified, and their full-text versions were obtained (Table S2).

Articles with one or more of these criteria were excluded from the meta-analysis: (a) acute interventions or measurements; (b) descriptive studies; and (c) indirect measures of executive function.

### 2.3. Data Extraction Process

Two independent authors extracted the data according to a previously established protocol. A third reviewer discussed the study data if differences or inconsistencies were found until an agreement about the data validity was made. The following information was collected: (1) author’s name and year of publication; (2) sample size and gender of participants; (3) origin of the sample (e.g., sports club or school); (4) socioeconomic status; (5) subjects’ age; (6) intervention program characteristics, in terms of program duration, frequency, session organisation, and length, and other relevant background information provided by the authors; (7) participants’ sport experience; (8) additional participants’ regular physical activity during the intervention program, and sport(s) included in the intervention program; (9) dimensions of executive function assessed and the instruments used; and (10) the limitations, suggestions, applications, and conclusions described in the studies.

All calculations were conducted using a Microsoft Excel (Microsoft, Redmond, WA, USA) spreadsheet containing data extracted from each publication. Review Manager (RevMan) version 5.3.5 was used for all the statistical analyses’ forest plots. The Cochran Q statistic [78] was used to assess heterogeneity between studies. Heterogeneity is a measure of the differences in main effects between studies. Additionally, I^2^ statistics were used to evaluate heterogeneity (I^2^ > 50%).

The effects of sports programs on executive function were calculated for each included study, following coding of the pre-to-post changes and standard deviations (SD) of both groups. The standardised mean difference (SMD) was calculated by subtracting baseline and post-intervention values of executive function measures. Data were required to take these forms: (a) the mean and SDs (pre- and post-intervention); (b) 95% confidence interval (CI) data for pre- to post-intervention changes for each group; or when this was unavailable, (c) actual *p*-values for pre- to post-intervention changes for each group; or, if only the level of statistical significance was available, (d) default *p*-values (e.g., *p* < 0.05 becomes *p* = 0.49, *p* < 0.01 becomes *p* = 0.0099, and *p* when not significant becomes *p* > 0.05). Random-effects inverse variance (IV) was used with the measurement of the effect of SMD.

The analysis of ES was conducted with a random-effects model estimated using the DerSimonian and Laird method [79]. A random-effects model is incorporated when the assumption is that the data demonstrated effects across studies that are randomly situated around a central value. Forest plots were generated to demonstrate the study-specific pre- to post-intervention effects on the executive function, or one of their main categories’ differences and ESs within the respective 95% CIs. Combining estimates then allowed for the assessment of a pooled effect. The reciprocal sums of two variances were accounted for, including the estimated variance associated with the study and the estimated variance component due to the variation between studies. A sensitivity analysis was conducted to identify highly influential studies that might have biased the analysis.

The study-specific weight was derived as the inverse of the square of the respective standard errors. ESs of ≤ 0.2, ≤ 0.5, ≤ 0.8, and ≥ 0.8 were considered trivial, small, moderate, and large, respectively [80].

## 3. Results

The flow diagram of the article search and selection is depicted in Figure 1, from the systematic search to inclusion.

### 3.1. Study Selection

The preliminary search yielded 3.265 relevant abstracts and citations. The full texts of 33 articles were deemed to meet the inclusion criteria. These 25 studies were rejected for this meta-analysis due to the reasons that can be seen in Figure 1. Finally, eight studies with 424 subjects met the inclusion criteria (Table 1) [25,26,27,28,29,81,82,83]. The experimental design of the included studies was a longitudinal study with pre- and post-intervention measurements. Of the 8 studies, working memory was registered in six studies [27,28,29,81,82,83], inhibitory control in five studies [25,27,28,29,81], and cognitive flexibility in six studies [26,27,28,29,81,82].

### 3.2. Quality Assessment

Table 2 shows the results of the methodological quality assessment for the eight included studies. The included studies ranged from a score of 3 to 6 points indicating poor or moderate quality [84]. The following study weaknesses were noted: all studies failed to blind participants or researchers to the intervention and had inadequate or absent randomisation [25,26,27,28,29,81,82], different group values at baseline [26,27], and poor compliance [27,81,82].

The PEDro database rapidly identifies which of the known or suspected randomised clinical trials (i.e., RCTs or CCTs) archived on the database are likely to be internally valid (criteria 2–9) and could have sufficient statistical information to make their results interpretable (criteria 10–11). An additional criterion (criterion 1) that relates to the external validity (or “generalizability” or “applicability” of the trial) has been retained so that the Delphi list is complete, but this criterion will not be used to calculate the PEDro score reported on the PEDro website.

### 3.3. Study Characteristics

Participants’ ages ranged from 8.8 ± 1.1 years to 17.5 ± 1.8 years. Six of the eight included studies incorporated both boys and girls in their sample; however, one incorporated girls exclusively [29] and one incorporated boys exclusively [83]. The intervention programs consisted of a sports program including one or several of the following sports: soccer, tennis, taekwondo, judo, floorball, basketball, handball, and athletics. The total duration of the programs ranged from 6 weeks to 1 year, with a frequency of 1–5 sessions per week and 30–90 min per session. Three of the studies were conducted in sports clubs, while the rest were conducted in the school context. One study incorporated novice players who had never participated in previous sports programs; three studies included participants with 3–4 years of experience in sport (sailing, tennis, and judo); and four studies did not report information related to participants’ previous experience (Table 3).

### 3.4. Assessment of Bias

**Figure 2:**
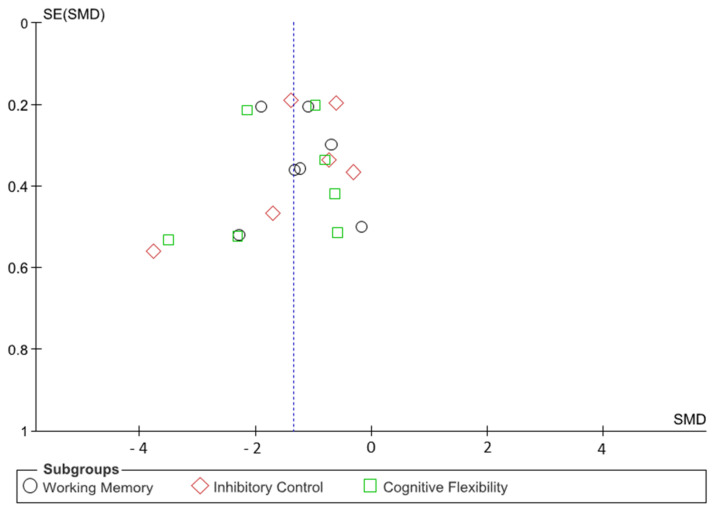
A funnel plot of the included studies to assess the potential risk of bias.

The authors did not detect any publication bias or heterogeneity in this meta-analysis. The funnel plot reveals that most data points within the plot are within the funnel, indicating that bias and between-study heterogeneity do not exist. If bias did exist, the data points would produce results outside of the reverse funnel, denoting asymmetry and bias (Figure 2).

### 3.5. Effects of Sports Activities on Executive Function

**Figure 3:**
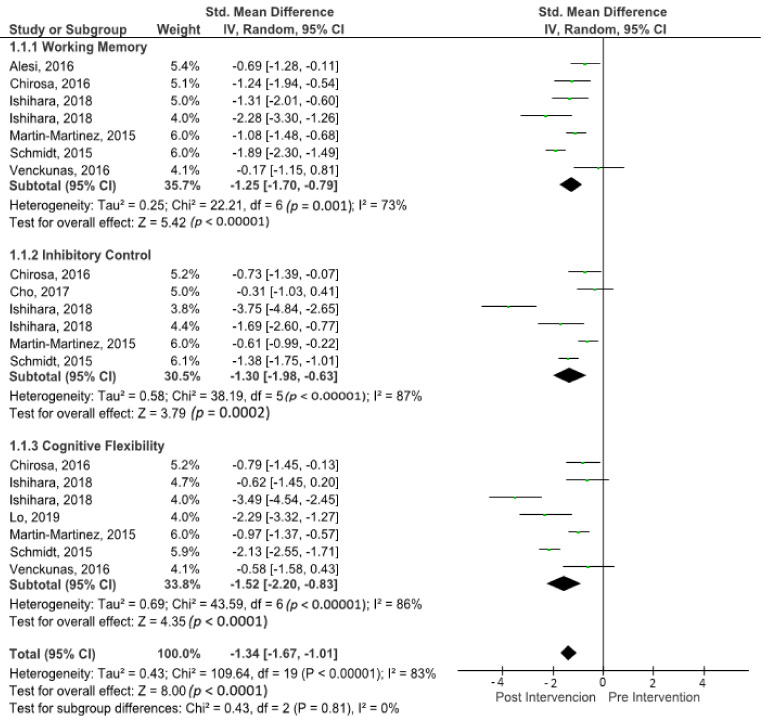
Forest plot of the effects on executive function. The vertical line indicates the overall estimate of the combined studies’ Scheme 95. CI, squares indicate estimates, square size is proportional to sample size, and rhombus indicates meta-analytically pooled estimates’ 95% CI. IV = inverse variance.

The outcomes for executive function are shown in the forest plot in Figure 3. The difference in executive functions between pre- and post-intervention measurements was assessed via a meta-analysis of all included studies. The three main categories of executive function were separated into different subgroup analyses. Due to potential heterogeneity, a random-effects model was incorporated with I^2^ and used to assess executive function measures. There was significant heterogeneity detected in all eight studies included in the meta-analysis (I^2^ = 83%). A large effect was observed when a random-effects analysis was applied for executive function outcomes (ES −1.34; 95% CI −1.67; −1.01; *p* < 0.00001).

### 3.6. Effects of Sports Activities on Working Memory

**Figure 4:**
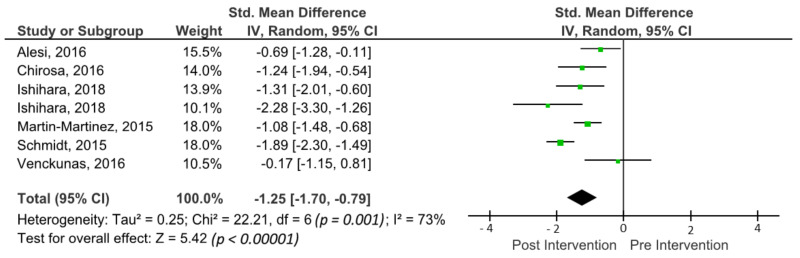
Forest plot of effects on working memory. The vertical line indicates the overall estimate of combined studies’ standardised mean effect size. The horizontal line indicates 95% CI, squares indicate estimates, square size is proportional to sample size, and rhombus indicates meta-analytically pooled estimates’ 95% CI. IV = inverse variance.

The outcomes for working memory are shown in the forest plot in Figure 4. The forest plot contains the SMD and corresponding CIs for working memory gain, as well as the overall effect test and heterogeneity analysis of the working memory in the experimental groups of the included studies. The pooled mean ES estimating working memory comprised seven sports groups from six studies. There was significant heterogeneity detected in this subgroup (I^2^ = 73%). When a random-effects analysis was applied, a large effect was observed in the post-treatment measurement for working memory outcomes (ES −1.25; 95% CI −1.70; −0.79; *p* < 0.0001).

### 3.7. Effects of Sports Activities on Inhibitory Control

**Figure 5:**
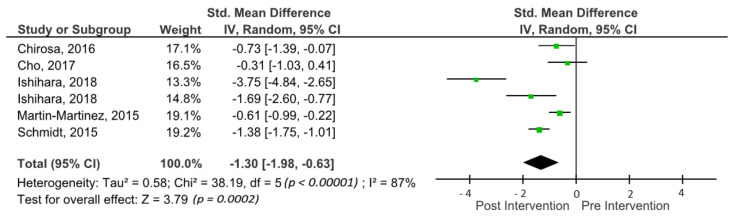
Forest plot of effects on inhibitory control. The vertical line indicates the overall estimate of combined studies’ standardised mean effect size. The horizontal line indicates 95% CI, squares indicate estimates, square size is proportional to sample size, and rhombus indicates meta-analytically pooled estimates’ 95% CI. IV = inverse variance.

The outcomes for inhibitory control are shown in the forest plot in Figure 5. The forest plot contains the SMD and corresponding CIs for inhibitory control measurements, as well as the overall effect test and the heterogeneity analysis of the inhibitory control in experimental groups of the included studies. The pooled mean ES estimating inhibitory control comprised six sports groups from five studies. There was significant heterogeneity detected in this subgroup (I^2^ = 87%). When a random-effects analysis was applied, a large effect was observed in the post-treatment measurement for inhibitory control outcomes (ES −1.30; 95% CI −1.98; −0.63; *p* < 0.00001).

### 3.8. Effects of Sports Activities on Cognitive Flexibility

**Figure 6:**
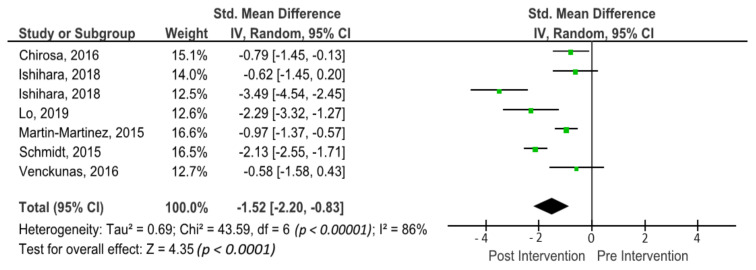
Forest plot of effects on cognitive flexibility. The vertical line indicates the overall estimate of the combined studies’ standardised mean effect size. The horizontal line indicates 95% CI, squares indicate estimates, square size is proportional to sample size, and rhombus indicates meta-analytically pooled estimates’ 95% CI. IV = inverse variance.

The outcomes for cognitive flexibility are shown in the forest plot in Figure 6. The forest plot contains the SMD and corresponding CIs for cognitive flexibility gain, as well as the overall effect test and heterogeneity analysis of the cognitive flexibility in the experimental groups of the included studies. The pooled mean ES estimating cognitive flexibility comprised seven sports groups from six studies. There was significant heterogeneity detected in this subgroup (I^2^ = 86%). When a random-effects analysis was applied, a large effect was observed in the post-treatment measurement for cognitive flexibility outcomes (ES −1.52; 95% CI −2.20; −0.83; *p* < 0.00001).

## 4. Discussion

The results obtained in the present meta-analysis show that: (I) while the study of physical activity and executive function is widely studied, the effects of sport on executive function is an emerging line of investigation that requires methodological quality improvements for future research (Table 2); (II) sports programs for healthy children and adolescents have a significant effect on the executive function of the participants. This highlights the role of sport as an effective way to enhance the development of executive function in children. These interventions can be performed in multiple contexts, either by preserving the original structure and integrating these interventions [25,26,83,85], by using the working method based on reduced games [28,29] or by generating enriched sports programs that are fully centred around cognitive development [27].

Compared to previous meta-analyses, this study and these results offer innovative findings based upon the type of intervention used; where other studies focused on physical exercise interventions, this study focuses exclusively on sport interventions [9,10,11,12,13]. Complex and varied movements, requiring adaptation to a dynamic environment, have been shown to improve both the quantity and quality of executive functioning and cognitive performance. In addition, it has been suggested that these activities should be exciting, cognitively challenging, and encourage emotional and social development [65,86,87,88]. Sports meet these characteristics and promote personal commitment, providing opportunities and experiences to achieve personal progress, thus breeding confidence [15,16,17,30]. The control of these variables in the design of these programs is highly relevant. Alesi et al. [83] and Schmidt et al. [27] explicitly incorporate these elements as part of their intervention programs, which are both based on group sports (football, floorball, and basketball).

A large body of evidence supports the close relationship between open-skill sports and executive functions, both in team sports, such as football, basketball, handball, etc., [16,20,21,22,23,27,28,29,83,89,90,91,92,93] and in individual sports, such as tennis [24,82,94,95,96] or combat sports, such as judo, karate, and taekwondo [26,30,97,98]. Team sports are particularly emphasised due to the high level of uncertainty caused by the fluid interaction between both teammates and opponents, providing an additional dimension that challenges and tests executive function compared to individual sports [16,23,28,29,93,99,100]. Martial arts are also recognised as a promising alternative for the improvement of executive functions, especially inhibitory control and cognitive flexibility [25,26,30,31,96,100].

The present study did not include results from studies with indirect or global measurements of executive functions [24,30,83] since the results were difficult to compare within the main dimensions of the included studies.

### 4.1. Working Memory

Working memory is a key aspect that enables the temporary storage and manipulation of information to perform task-oriented behaviours in a variety of cognitive contexts [7]. The results of this meta-analysis show that sports are beneficial for working memory in children and adolescents, showing a significant effect of a large magnitude (ES −1.25; 95% CI −1.70; −0.79; *p* < 0.0001). These results are meaningful when compared with previous meta-analyses where acute and chronic effects were assessed on working memory in children and adolescents. Studies such as De Greeff et al. [9] with an ES: 0.36; Liu et al. [10] with an ES: −0.54 and Alvarez-Bueno et al. [13] with an ES: 0.14, found small or moderate effect sizes for chronic physical activity interventions.

Furthermore, other studies suggest no significant effect on working memory when chronic exercise interventions are performed in children and adolescents [12]. It has not been possible to relate the large effect size found in the present meta-analysis for working memory to any variable of the sport-based programs. However, it can be observed the longer the duration and frequency of the intervention, the larger the effects (ES: −2.28) [85]. Additionally, Liu et al. [10] highlight the role of exercise type, reporting differences in ES in favour of open motor skills (SMD = −0.72, 95% CI −0.93 to −0.43) versus closed motor skills (SMD = −0.31, 95% CI −0.57 to −0.25). In this regard, five of the six studies included in the present meta-analysis for working memory included open-ability sports [27,28,29,83,85].

### 4.2. Inhibitory Control

Inhibitory control is the skill of managing attention, behaviour, thoughts, and emotions in order to overcome strong internal bias or distracting external stimuli, enabling us to make the most appropriate response in a specific context [6,101,102]. The results obtained in this meta-analysis showed significant ES inhibition control in healthy children and adolescents (ES −1.30; 95% CI −1.98; −0.63; *p* < 0.00001). The results of this study stand out from previous studies, which have found small effect sizes for chronic exercise on inhibitory control in this population, such as the meta-analyses by Liu et al. [10] (ES: −0.30); Xue et al. [12] (ES: 0.26) and Álvarez-Bueno et al. [13] (ES: 0.26) among others. These results oppose studies such as De Greeff et al. [9], where no significant effects on inhibitory control were found after the application of physical activity programs in children (ES: 0.19; 95% CI = −0.04, 0.42). This may be because this executive function domain was in the early stages of development at this age [103,104,105].

Previous studies support the theory that cognitively engaging and challenging exercises are particularly important for improving inhibitory control in children and adolescents [74,106]. However, there is not enough research suggesting which variables of the sports training process are the most relevant to improving inhibitory control. If the results obtained by the included studies are analysed, it can be observed that the longer the duration of an intervention, the greater the ES associated with it [85]. There is no clear relationship between ES and the particular characteristics of the intervention programs in relation to the other variables.

### 4.3. Cognitive Flexibility

Cognitive flexibility enables the ability to shift attention between multiple tasks according to the demands of the environment or previously established priorities [6]. In relation to the reported ES, the present study shows large effects in post-intervention measurements (ES −1.52; 95% CI −2.20; −0.83; *p* < 0.00001), which showed the largest ES compared to the other two dimensions analysed in the present study (working memory and inhibitory control). Previous meta-analyses including children and adolescents agree with these findings by showing significant effects on cognitive flexibility after implementing longitudinal physical exercise interventions; however, their effect sizes are small. Regarding the above, De Greeff et al. [9] found ES: 0.18, Liu et al. [10] found ES: −0.34, while Álvarez-Bueno et al. [13] found ES: 0.11. One of the studies reviewed found no significant effect of chronic exercise interventions on cognitive flexibility in this population [12]. The present study found the same pattern on the three executive function dimensions. It was found that the longer the length and the higher the frequency of the sport-based program, the larger the reported effect size (ES: −3.49) [85]. This could highlight the importance of these variables on the three main dimensions of executive functioning. It would be interesting to explore the effect of the length and frequency of the programs on the main outcomes.

Further research is needed to analyse which characteristics of sports have a strong influence on the improvement of executive function categories. However, the current literature provides substantial evidence that sports provide an optimal context for the development of executive function in children and adolescents [26,83,85]. In addition, it is necessary to design tasks efficiently, track personal performance, evaluate new information, and encourage self-control, self-correction, and self-improvement [24,26,30].

### 4.4. Strengths, Limitations, and Suggestions for Future Research

The main findings obtained from the detailed analysis of the included studies highlight some key themes. Sport-based programs result in significant executive function improvements in children and teenagers; however, more studies are required to provide more consistent results. Open-skill sports provide a large number of stimuli to be inhibited, a large amount of information to be processed, and the requirement of choosing an option with that input [26,83,85]. In future research, it would be interesting to control variables related to the instructors, such as the learning style or the feedback provided to the participants.

Considering the limited number of studies included in this meta-analysis, the interpretation of the results must consider certain limitations: (1) On one hand, no previous registration of the review protocol has been made in PROSPERO EQUATOR or any another similar platform. (2) It was not possible to assess the effect of sport on high-level executive functions, such as reasoning or problem resolution. These variables may be explored and taken into account to complement the information provided in this study. (3) Most of the studies analysed do not report information on the socio-economic level of their participants, while some of them present missing information on other relevant variables, such as the intensity of the sports activities performed. (4) No follow-up was implemented, so the maintenance and generalisation of improvements beyond the duration of the programs are unknown.

Future research should compare the effect of different sports modalities such as team sports or combat sports. It would also be interesting to evaluate different teaching styles that create challenging environments. In addition, it would be interesting to improve the methodological quality of the studies by providing a detailed description of the intervention programs or a follow-up to describe the duration of the effects obtained. Furthermore, it is considered important to report the socio-economic status of the participants and other physical activities performed during the intervention period.

## 5. Conclusions

The current meta-analysis demonstrates that sports may have a positive effect on children’s and teenagers’ executive function. In addition, it suggests that organised sports might be a better option for executive function development than simply increasing physical activity. With sport interventions, large ESs were found in all executive function categories, in contrast to the small or medium ESs reported by previous meta-analyses, which focused on physical activity. This research encourages the use of sports programs for children due to the fact that they can provide the physical activity experiences needed to form healthy habits and also bring health benefits, such as executive function development, especially with regard to cognitive flexibility. Social mediators, such as parents, teachers, and local governments, should approach sports practice as being a route for promoting universal development in children, garnering both physical and cognitive benefits.

Nevertheless, not enough evidence has been found to recommend a specific sport modality over others. To better understand the effects of sports programs, a rigorous study design is necessary. Future research should report the volume, intensity, and types of exercises performed. Additionally, the effects of different sports modalities should be explored.

## Figures and Tables

**Table 1:** Subjects’ characteristics from the included studies.

Reference	Country	N	Sex	Age (Years)	Socioeconomic Status
**Alesi et al., 2016**	Italy	44; EG: 24, CG: 20	44 M	EG: 8.8 (1.1); GC: 9.3 (0.9)	Medium
**Chirosa et al., 2016**	Spain	39; EG: 20, CG:19	39 F	15.41 (0.50)	Not reported
**Cho et al., 2017**	Korea	30; EG: 15, CG:15	18 M/12 F	EG: 11.20 (0.77); CG: 11.33 (0.72)	Not reported
**Ishihara et al., 2017**	Japan	32; EG:19, CG: 13	20 M/12 F	9 (1)	Not reported
**Lo et al., 2019**	China	29; EG:14, CG: 15	22 M/7 F	EG: 13.36 (1.15); CG: 13.47 (1.24)	Not reported
**Martín-Martínez et al., 2015**	Spain	54	14 M/40 F	15.35 (0.48)	Not reported
**Schmidt et al., 2015**	Switzerland	181; EG1: 69; CG: 55	82 M/99 F	11.35 (0.60)	Not reported
**Venckunas et al., 2016**	Lithuania	18; EG: 8, CG: 10	14 M/4 F	EG: 17.0 (1.1); GC: 17.5 (1.8)	Not reported

N: number of subjects; M: male; F: female; EG: experimental group; CG: control group.

**Table 2:** PEDro Scale of the studies included.

Study	1	2	3	4	5	6	7	8	9	10	11	Total
**Alesi et al., 2016**				✓				✓	✓	✓	✓	5
**Chirosa et al., 2016**		✓		✓				✓	✓	✓	✓	6
**Cho et al., 2017**	✓	✓		✓				✓	✓	✓	✓	6
**Ishihara et al., 2017**				✓						✓	✓	3
**Lo et al., 2019**								✓	✓	✓	✓	4
**Martín-Martínez et al., 2015**		✓		✓				✓	✓	✓	✓	6
**Schmidt et al., 2015**		✓								✓	✓	3
**Venckunas et al., 2016**				✓						✓	✓	3

**Table 3:** Intervention characteristics of the included studies.

Studies	Sport Training	Sport	Intervention Length	Weekly Frequency	Session Length	Intensity
**Alesi et al., 2016**	Exercises involving individual skills, one-on-one situations and three-on-three and five-on-five games.	Football	6 months	2/week	75 min	Not reported
**Chirosa et al., 2016**	1st session: 6 small-sided games 3 × 3 (2 football, 2 basketball, and 2 handball without a goalkeeper). 2nd session: 3 small-sided games 3 × 3 (1 football without a goalkeeper, 1 basketball and 1 handball without a goalkeeper).	Football, basketball, and handball	8 weeks	3/week	1º session: 60 min 2º session: 30 min	(1) Heart Rate: 175.68 ± 11.39 bpm (2) RPE 13.20 ± 1.53
**Cho et al., 2017**	Physical fitness training, Poomsae, Kicking, Gymnastics with TKD movements.	Taekwondo	16 weeks	5/week	60 min	RPE: 11–15
**Ishihara et al., 2017**	Tennis lessons (unspecified exercises).	Tennis	12 months	EG1: 1/weekEG2: 4/week	Not reported	MVPA
**Lo et al., 2019**	Falling techniques, throwdown, pushdown, hook down, and Randori (Judo fighting).	Judo	8 weeks	3/week	90 min	Not reported
**Martín-Martínez et al., 2015**	1st session: 6 small-sided games 3 × 3 (2 football, 2 basketball, and 2 handball without a goalkeeper). 2nd session: 3 small-sided games 3 × 3 (1 football without a goalkeeper, 1 basketball and 1 handball without a goalkeeper).	Football, basketball, and handball	8 weeks	2/week	1º session: 60 min 2º session: 30 min	(1) Heart Rate: 175.96 ± 10.26 bpm (2) RPE: 13.36 ± 1.39
**Schmidt et al., 2015**	EG1 (high cognitive engagement, high physical exertion): team games. EG2 (high cognitive engagement, high physical exertion): aerobic exercises.	Floorball and basketball	6 weeks	2/week	45 min	MVPA
**Venckunas et al., 2016**	High-intensity exercise (200–1000 m intervals) time increased from only 12 min at week 1 to 30 min by week 7.	Track and Field	7 weeks	4/week	Increased from 42 min to 90 min at the end.	Heart Rate during exercise: 190 bpm Heart Rate during rest: 130 bpm

Bpm: beats per minute; RPE: rate of perceived exertion; MVPA: moderate to vigorous physical activity.

## Supplementary Materials

The following are available online at https://www.mdpi.com/article/10.3390/brainsci11060755/s1, Table S1: Search Strategy, Table S2: Inclusion Criteria Assessment.
